# Correlates of sedentary behaviour among adolescents and adults with hazardous, harmful or dependent drinking in South Africa

**DOI:** 10.4102/sajpsychiatry.v25i0.1217

**Published:** 2019-07-04

**Authors:** Karl Peltzer, Nancy Phaswana-Mafuya, Supa Pengpid

**Affiliations:** 1 Department for Management of Science and Technology Development, Ton Duc Thang University, Ho Chi Minh City, Vietnam; 2 Faculty of Pharmacy, Ton Duc Thang University, Ho Chi Minh City, Vietnam; 3 Research and Innovation Office, North West University, Potchefstroom, South Africa; 4 ASEAN Institute for Health Development, Mahidol University, Salaya, Thailand

**Keywords:** Sedentary behaviour, Problem drinking, Correlates, National survey, South Africa

## Abstract

**Background:**

There is lack of information on the correlates of sedentary behaviour among persons with alcohol use disorders. The study aimed to examine socio-demographic and health correlates among adolescents and adults with hazardous, harmful or probable dependent alcohol use (= problem drinking).

**Method:**

Data from the cross-sectional South African National Health and Nutrition Examination Survey (SANHANES-1) 2011–12 were analysed. From a total sample of 15 085 persons aged 15 years and older, 2849 adolescents and adults (mean age = 37.1 years, standard deviation [s.d.] = 15.1) were identified as problem drinkers, based on the Alcohol Use Disorders Identification Test-Consumption (AUDIT-C). Multivariable logistic and linear regression were used to determine the associations between socio-demographic characteristics, health variables and high sedentary behaviour (≥8 h/day) and total minutes of sedentary behaviour a day.

**Results:**

The prevalence of high sedentary behaviour (≥ 8 h/day) was 11.9% overall (11.9% among men and 12.1% among women), and the mean (s.d.) duration of sedentary behaviour was 263 (169) min/day. In bivariate analysis, older age, population group, functional disability, cognitive impairment, having hypertension, having had a stroke and posttraumatic symptoms were correlated with high sedentary behaviour. In adjusted logistic regression analysis, older age and being Indian or Asian were positively, and having been diagnosed with angina was negatively, associated with high sedentary behaviour. In linear regression analysis, older age, not employed and having had a stroke were positively, and being of mixed race and having angina were negatively, associated with total minutes (up to 960 min/day) of sedentary behaviour in a day.

**Conclusion:**

The study provides socio-demographic and health correlates of sedentary behaviour among problem drinkers. This information can guide possible future interventions in reducing sedentary behaviour among problem drinkers.

## Introduction

Globally, harmful alcohol use ranks 8th among the 79 largest contributors to global disability-adjusted life-years (DALYs).^1^ In South Africa, alcohol harm was responsible for an estimated 7.0% of total DALYs and 7.1% of all deaths.^2^ Risky alcohol consumption (e.g. hazardous or harmful drinking 9.0% and binge drinking 14.1%) has been found to be prevalent in South Africa.^3,4^ Individuals who are hazardous, harmful or dependent alcohol drinkers (= problem drinkers) are at higher risk of physical illness (infectious diseases such as tuberculosis and non-communicable diseases such as cardiovascular disease and diabetes) and mental illness and mortality.^5,6^

Considering the limitations of various biological and psychological treatment approaches for alcohol use disorders, Vancampfort et al.^7^ stress the need for new interventions, such as physical activity, that may help in reducing alcohol intake as well as physical and mental comorbidity.^8^

‘Sedentary behavior refers to any waking behavior characterized by an energy expenditure ≤ 1.5 metabolic equivalent (MET) while in a sitting or reclining posture’.^9^ Among youth and adults significant evidence exists that sedentary behaviour, independently from the levels of physical activity, is associated with morbidity (hypertension, total cholesterol, cardiometabolic risk, type 2 diabetes and metabolic syndrome) and mortality.^10,11,12^ In a systematic review in youth, adult and older adult populations,^13,14,15,16,17^ correlates for sedentary behaviour included increasing age, gender, education, employment status, income, obesity, smoking status, poor physical health and poor mental health (anxiety, depressive symptoms).

Considering the importance of sedentary behaviour interventions, that may differ in terms of correlates from physical activity interventions,^13^ there is a special need to target sedentary behaviour in special groups or vulnerable populations such as those who are problem drinkers.^5^ Because associations with sedentary behaviour among problem drinkers may differ to those in the general population,^14^ it is relevant to study correlates of sedentary behaviour among problem drinkers separately. Therefore, the study aimed to examine socio-demographic and health correlates among adolescents and adults with hazardous, harmful or probable dependent alcohol use (= problem drinking) in a national survey in South Africa.

## Methods

### Sample and procedure

The first South African National Health and Nutrition Examination Survey (SANHANES-1) is a cross-sectional and multistage population-based household health survey conducted in 2012.^18^ Participants were interviewed with a questionnaire on socio-demographic and health variables after their informed consent was obtained. The study protocol was approved by the research ethics committee (REC) of the HSRC (REC 6/16/11/11).

The survey response rate of participants was 92.6%.

### Measures

Sedentary behaviour was measured with two questions on the time sitting or reclining in the past 7 days during a usual weekday and usual weekend day.^19,20^ Responses were recorded in hours and minutes. Sedentary times were truncated at 960 min/day (16 h). Times spent sedentary were summed to calculate the total minutes per day in the last 7 days.^20^ High sedentary behaviour was defined as ≥ 8 h/day.^10,21^

Problem drinking was assessed with the three-item Alcohol Use Disorders Identification Test-Consumption (AUDIT-C), with scores of 3 or more in women and 4 or more in men indicating hazardous, harmful or dependent alcohol use (or problem drinking)^22^ (Cronbach’s alpha 0.89).

Socio-demographic information included sex, age, population group, employment and residential status.

Self-rated health was assessed with the item: ‘In general, how would you rate your health today?’^18^ Responses were dichotomised into having ‘good health’ (= 1: very good, or 2: good) or ‘poor health’ (= 3: moderate, 4: bad or 5: very bad).

Functional disability was assessed with the item: ‘Overall in the last 30 days, how much difficulty did you have with work or household activities?’ Response options ranged from 1 = none, 2 = mild, 3 = moderate, 4 = severe to 5 = extreme.^18^ Functional disability was defined as moderate to extreme difficulties.

Cognitive impairment was assessed with two items:

How much difficulty did you have with concentrating or remembering things?

How much difficulty did you have in learning a new task (e.g. learning how to get to a new place, learning a new game, learning a new recipe)?^18^

Response options range from 1 = none to 5 = extreme. The two items were summed and cognitive impairment was defined as moderate to severe (scores 6–10).

Current tobacco use was assessed with two questions on daily or less than daily tobacco smoking and use of other tobacco products.^18,23^

Fruit consumption: ‘How many fruits do you usually eat per day?’ Vegetable consumption: ‘How many portions of vegetables, excluding potatoes, do you usually eat per day?’ Response options were 1 = 4 or more per day, 2 = 1–3 per day, 3 = not every day, but 4 or more a week, 4 = not every day, but less than 4 per week, and 5 = none.^18^

Handwashing hygiene behaviour was assessed with the question: ‘How often do you wash your hands before eating?’ Response options ranged from 1 = always to 4 = never.^18^

Bodily pains were measured with the question: ‘How much bodily aches or pains did you have?’ Response options ranged from 1 = none to 5 = extreme or cannot do.^17^ Bodily pains were defined as ‘moderate, severe, extreme/can’t do’.

Physical ill-health conditions were assessed with the question: ‘Has a doctor or nurse or health worker at a clinic or hospital told you that you have had any of the following conditions? High blood pressure, stroke, heart disease, a heart attack or angina (chest pains), high blood cholesterol, high blood sugar or sugar diabetes, and tuberculosis’.^18^

Visual difficulties were assessed with two questions asking about the difficulty of near and distant vision. Response options ranged from 1 = none to 5 = extreme or cannot do.^18^ Both questions were summed and visual difficulty was defined as ‘moderate, severe, extreme/can’t do’ (scores 6–10).

Hearing difficulties were assessed with two items about the difficulty in ‘hearing someone talking on the other side of the room in a normal voice’ and ‘hearing what is said in a conversation with one other person in a quiet room’. Response options ranged from 1 = none to 5 = extreme or cannot do.^18^ Both questions were summed and hearing difficulty was defined as ‘moderate, severe, extreme/can’t do’ (scores 6–10).

Sleep problems were measured with the item: ‘How much of a problem did you have with sleeping, such as falling asleep, waking up frequently during the night, or waking up too early in the morning?’ Response options ranged from 1 = none to 5 = extreme/cannot do. Sleeping problems were classified in response to this question as ‘moderate’, ‘severe’ or ‘extreme/cannot do’.^24^

Psychological distress in the past month was assessed with the 10-item Kessler 10 scale,^25^ and has been validated in South Africa.^26^ Response options ranged from 1 = never to 5 = all of the time and the total score is a summation of all the responses, with scores of 20 or more indicating mild, moderate or severe psychological distress.^25^ Cronbach’s alpha for the Kessler 10 scale was 0.91 in this sample.

Posttraumatic stress disorder (PTSD) was assessed with the 17-item Davidson Trauma Scale (DTS) that assesses all primary DSM-IV symptoms of PTSD related to intrusion, avoidance and hyperarousal symptoms. Participants were considered to have PTSD ‘if they score at least one -re-experiencing, three avoidance/numbing and two hyper-arousal phenomena at a frequency of at least twice in the previous week’.^27^ In this analysis, we used any of the three symptom criteria (Cronbach’s alpha 0.94).

### Data analysis

Data were analysed with STATA software version 13.0 (Stata Corporation, College Station, TX, USA). Descriptive statistics were calculated for the proportions of the study variables. Pearson chi-square statistics was used to test for differences in proportions. We used multivariable logistic and linear regression to determine the associations between socio-demographic characteristics, health variables and high sedentary behaviour (≥ 8 h/day) and total minutes of sedentary behaviour a day. No collinearity was detected. Missing data were excluded from the analysis. All results were adjusted for the multistage sampling design.

## Ethical consideration

The study protocol was approved by the research ethics committee (REC) of the HSRC (REC 6/16/11/11).

## Results

### Sample characteristics

From a total sample of 15 085 persons aged 15 years and older, 2849 adolescents and adults (mean age = 37.1 years, standard deviation [s.d.] = 15.1) had been identified as hazardous, harmful or dependent drinkers (or problem drinkers). In the sample of problem drinkers, the prevalence of high sedentary behaviour (≥ 8 h/day) was 11.9% (11.9% among men and 12.1% among women), and the mean (s.d.) duration of sedentary behaviour was 263 (169) min/day.

Compared with individuals without hazardous, harmful or dependent drinking, the prevalence of high sedentary behaviour (13.7%) and the mean duration of sedentary behaviour (267 min/day) did not significantly differ from persons with hazardous, harmful or dependent drinking (11.9%) (*p* = 0.117) and (263 min/day) (*p* = 0.223). In the total sample, more men (70.4%) than women (29.6%) were hazardous, harmful or dependent drinkers (*p* < 0.001), and drinkers were younger than non-drinkers (*p* < 0.001). Table 1 describes the sample characteristics and prevalence of high sedentary behaviour among problem drinkers.

**TABLE 1:** Sample characteristics and prevalence of high sedentary behaviour among problem drinkers (*N* = 2849).

Variable	Sample		Sedentary behaviour (≥ 8 h/day)		
			No	Yes	Chi-square
	*N*	%	%	%	*p*
**Socio-demographic factors**					
**Age (years), *M* = 37.1 (s.d. = 15.1)**	-	-	-	-	-
15–24	721	23.1	24.1	16.8	0.037
25–44	1266	51.1	49.9	59.2	-
45–64	722	21.7	22.0	17.8	-
65+	135	4.0	4.0	6.2	-
**Sex**	-	-	-	-	-
Female	1015	70.4	70.8	70.4	0.918
Male	1834	29.6	29.2	29.6	-
**Population group**	-	-	-	-	-
Black African	1746	71.3	70.4	76.6	0.011
White people	186	13.6	14.1	9.3	-
Mixed race people	813	13.6	14.2	10.1	-
Indian or Asian	92	1.5	1.2	4.0	-
**Employment status**	-	-	-	-	-
Employed	1227	47.6	48.4	40.7	0.104
Not employed	1565	52.4	51.6	59.3	-
**Residence**	-	-	-	-	-
Rural	855	28.4	29.8	26.3	0.374
Urban	1994	71.6	70.2	73.7	-
**Health status**	-	-	-	-	-
Self-rated health status (poor)	684	22.9	22.8	26.8	0.315
Functional disability	284	9.2	8.4	15.3	0.009
Cognitive impairment	157	5.2	4.7	8.1	0.049
**Health behaviour**	-	-	-	-	-
Current tobacco use	1382	47.1	48.0	45.4	0.581
Fruits (less once/day)	1344	45.1	45.0	49.1	0.329
Vegetables (less once/day)	1266	43.8	43.3	47.2	0.374
Hand washing before meals (not always)	552	20.8	20.2	19.2	0.776
**Physical health**	-	-	-	-	-
Bodily pain	369	12.7	12.1	15.9	0.140
Ever had tuberculosis	246	8.5	8.2	9.6	0.532
Hypertension	468	14.7	14.1	18.4	< 0.001
High cholesterol	101	3.8	3.3	3.9	0.674
Diabetes	106	3.0	2.9	2.3	0.536
Stroke	55	1.8	1.3	4.9	< 0.001
Angina	104	4.1	4.1	4.9	0.633
Heart disease	58	1.7	1.6	2.8	0.187
Visual problems	145	4.9	4.5	7.4	0.145
Hearing problems	50	1.3	1.1	1.3	0.765
**Mental health**	-	-	-	-	-
Sleep problem	288	10.9	10.3	13.7	0.199
Psychological distress (20+)	508	17.2	16.4	22.5	0.069
PTSD any of three symptom criteria	267	11.2	11.4	13.2	0.008

PTSD, posttraumatic stress disorder; s.d., standard deviation.

### Associations with sedentary behaviour

In bivariate analyses, older age, population group, functional disability, cognitive impairment, having hypertension, having had a stroke and PTSD symptoms were correlated with high sedentary behaviour (see Table 1).

In adjusted logistic regression analysis, older age and being Indian or Asian were positively, and having been diagnosed with angina was negatively, associated with high sedentary behaviour, while in linear regression analysis older age, not employed and having had a stroke were positively, and being of mixed race and having angina were negatively, associated with total minutes of sedentary behaviour in a day (see Table 2).

**TABLE 2:** Associations of socio-demographic and health variables with sedentary behaviour levels among problem drinkers.

Variable	Logistic regression: Sedentary ≥ 8 h/day		Linear regression: Minutes per day sedentary	
	AOR	95% CI	*b*-Coefficient	95% CI
**Socio-demographic factors**					
**Age (years)**	-	-	-	-
15–24	1	Reference	-	Reference
25–44	2.44	1.44–4.13***	14.65	1.89–26.70*
45–64	1.42	0.72–2.81	16.70	1.52–30.60*
65+	2.43	1.01–6.15*	46.92	18.55–75.34***
**Sex**	-	-	-	-
Female	1	Reference	-	Reference
Male	1.10	0.75–1.64	7.19	−1.58–15.96
**Population group**	-	-	-	-
Black African	1	Reference	-	Reference
White people	0.84	0.36–1.94	−9.71	−33.06–13.66
Mixed race people	0.70	0.43–1.15	−26.08	−43.73–-9.07**
Indian or Asian	3.40	1.72–6.72***	−17.07	−48.16–14.21
**Employment status**	-	-	-	-
Employed	1	Reference	-	Reference
Not employed	1.38	0.69–2.74	28.77	14.86–42.68***
**Residence**	-	-	-	-
Rural	1	Reference	-	Reference
Urban	1.26	0.75–2.13	9.38	−16.88–35.63
**Health status**	-	-	-	-
Self-rated health status (poor)	1.03	0.60–1.78	1.17	−12.71–15.06
Functional disability	1.80	0.94–3.42	6.69	−13.71–27.13
Cognitive impairment	1.02	0.42–2.48	1.55	−6.18–9.29
**Health behaviour**	-	-	-	-
Current tobacco use	0.74	0.49–1.11	−3.22	−14.57–8.14
Fruits (less once/day)	1.07	0.69–1.65	7.93	−6.68–22.53
Vegetables (less once/day)	1.05	0.71–1.57	−4.92	−19.93–10.09
Hand washing before meals (not always)	0.96	0.56–1.67	10.00	−6.62–26.63
**Physical health**	-	-	-	-
Bodily pain	1.37	0.69–2.74	1.46	−14.81–17.75
Ever had tuberculosis	1.08	0.58–2.00	3.86	−12.88–20.55
Hypertension	1.59	0.91–2.78	14.25	−0.42–23.25
High cholesterol	0.67	0.21–2.16	3.31	−28.83–34.75
Diabetes	0.54	0.20–1.45	−13.78	−34.84–7.28
Stroke	2.94	0.81–10.71	44.17	10.39–77.59**
Angina	0.28	0.08–0.93*	−27.04	−50.75–-3.33*
Heart disease	1.12	0.32–3.92	−2.55	−36.21–31.11
Visual problems	1.29	0.58–2.84	−8.81	−35.22–17.95
Hearing problems	0.99	0.25–3.85	30.88	−26.53–88.30
**Mental health**	-	-	-	-
Sleep problem	0.94	0.44–2.02	−11.98	−29.71–4.63
Psychological distress (20+)	1.22	0.67–2.22	5.00	−14.52–24.52
PTSD any of three symptom criteria	0.97	0.55–1.69	2.29	−19.66–24.25

AOR, adjusted odds ratio; CI, confidence interval; PTSD, posttraumatic stress disorder.

*** *p* < 0.001;

** *p* < 0.01;

* *p* < 0.05.

## Discussion

This is the first study in Africa to investigate correlates of sedentary behaviour among problem drinkers. The investigation found that among problem drinkers (*N* = 2849, mean age = 37.1 years) the prevalence of high sedentary behaviour was 11.9% and the mean (s.d.) duration of sedentary behaviour was 263 (169) min/day; this did not differ significantly with non-problem drinkers. The sedentary behaviour duration among problem drinkers seems to be higher in this study than in a study among hazardous drinkers in six middle-income countries (with a higher mean age = 45.7 years), including South Africa, with a prevalence of 9% of high sedentary behaviour and a mean duration of sedentary behaviour of 216 min.^7^

This study found among problem drinkers that sedentary behaviour increased with age and that there were population group differences: Indian or Asian persons had a higher prevalence of high sedentary behaviour than other population groups, and back Africans had a higher total time of sedentary behaviour than the other, especially mixed race people, population groups. In the general adult population, older age was associated with higher sedentary behaviour.^13,14,15^ It is not clear as to why we found such racial or cultural differences, which needs further investigation. In a study among five different ethnic groups in the general population in the Netherlands, no significant ethnic differences were found in objectively measured sedentary behaviour.^28^

In our study, not being employed was a strong predictor of increasing minutes per day sedentary behaviour among problem drinkers. A similar correlation was found in a previous study in six middle-income countries, including South Africa.^7^ It is possible that having a job increases physical activity and may involve active transportation to go to work.^7^ In addition, problem drinking may be more prevalent in the unemployed, can cause unemployment and may reduce chances of getting employed.^7,29^ While in the study^7^ in six middle-income countries older adults living in urban areas were more likely to engage in sedentary behaviour, this study did not find any urban–rural differences.

In bivariate analyses, functional disability and cognitive impairment were associated with high sedentary behaviour among problem drinkers. In agreement with a previous study,^5^ this study also found an association between having had a stroke and spending more time in sedentary behaviour among problem drinkers. Contrary to expectation and previous studies,^30,31^ this study found a negative association between angina and sedentary behaviour. It is possible that the individuals with angina have been attending a cardiac rehabilitation programme that includes the promotion of physical activity. A previous review^32^ has shown that increasing physical activity can promote healthy ageing (functional ability), possibly also preventing or delaying functional disability and cognitive impairment. In a community survey in Cape Town, South Africa, stroke survivors were found to engage in a high amount of sedentary time.^33^ For people who have had a stroke to engage in physical activity may not be easy, but it would be important to develop possible ways of undertaking physical activity in this population.^7^ On the contrary, problem drinking increases the risk of getting a stroke.^5^

Our findings seem to show that helping problem drinkers to get employment may be a relevant strategy to decrease sedentary behaviour.^7^ An increase in physical activity may also improve the mental and physical health of the problem drinkers.^7^ Furthermore, one may need to consider socio-demographic factors such as age and population group and having chronic conditions, such as stroke, in designing interventions in problem drinkers. Strategies to combine interventions to decrease sedentary behaviour with decreasing problem drinking could be effective and should be tested.

## Study limitations

The study variables, such as sedentary behaviour, alcohol use and physical illness conditions, were self-reported, and the cross-sectional nature of the study limits our ability to establish causality. Longitudinal studies on sedentary behaviour among problem drinkers are warranted.

## Conclusion

This investigation found among problem drinkers that older age, being Indian or Asian, not employed and having had a stroke were positively, and being of mixed race and having been diagnosed with angina were negatively, associated with high sedentary behaviour and/or with total minutes of sedentary behaviour in a day. Findings provide information on possible future interventions that can help to reduce sedentary behaviour among problem drinkers.
